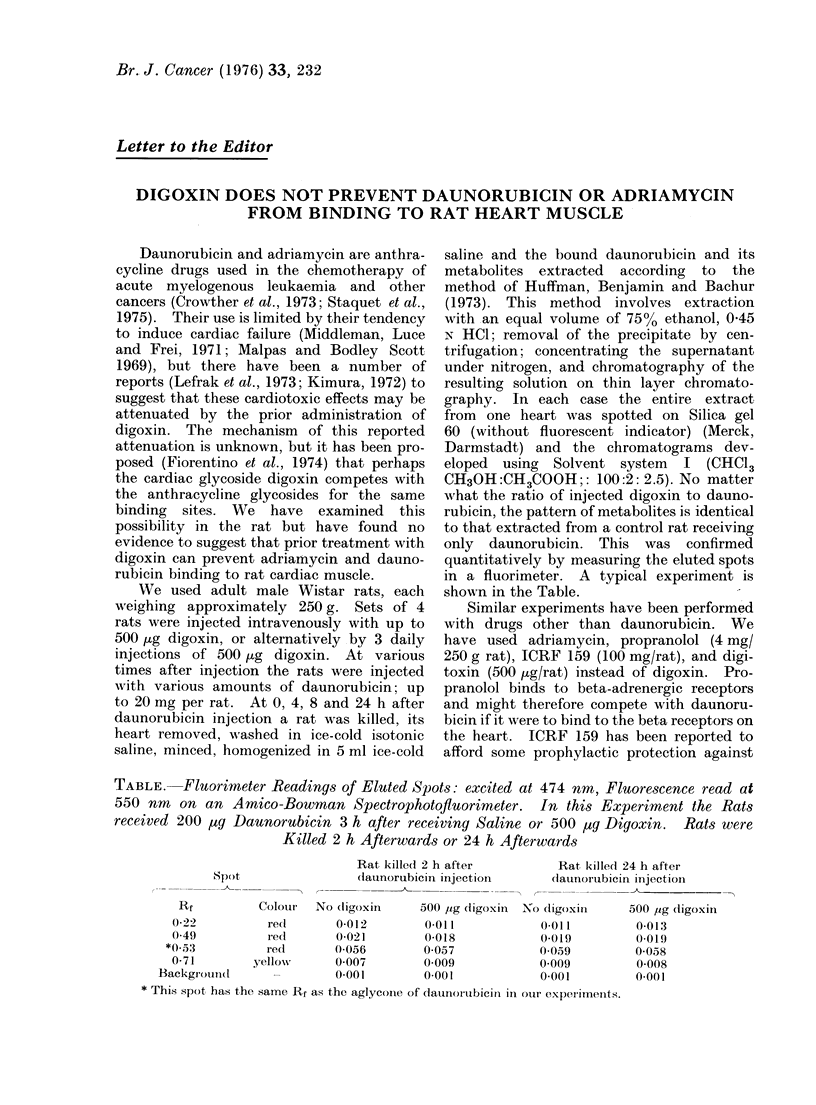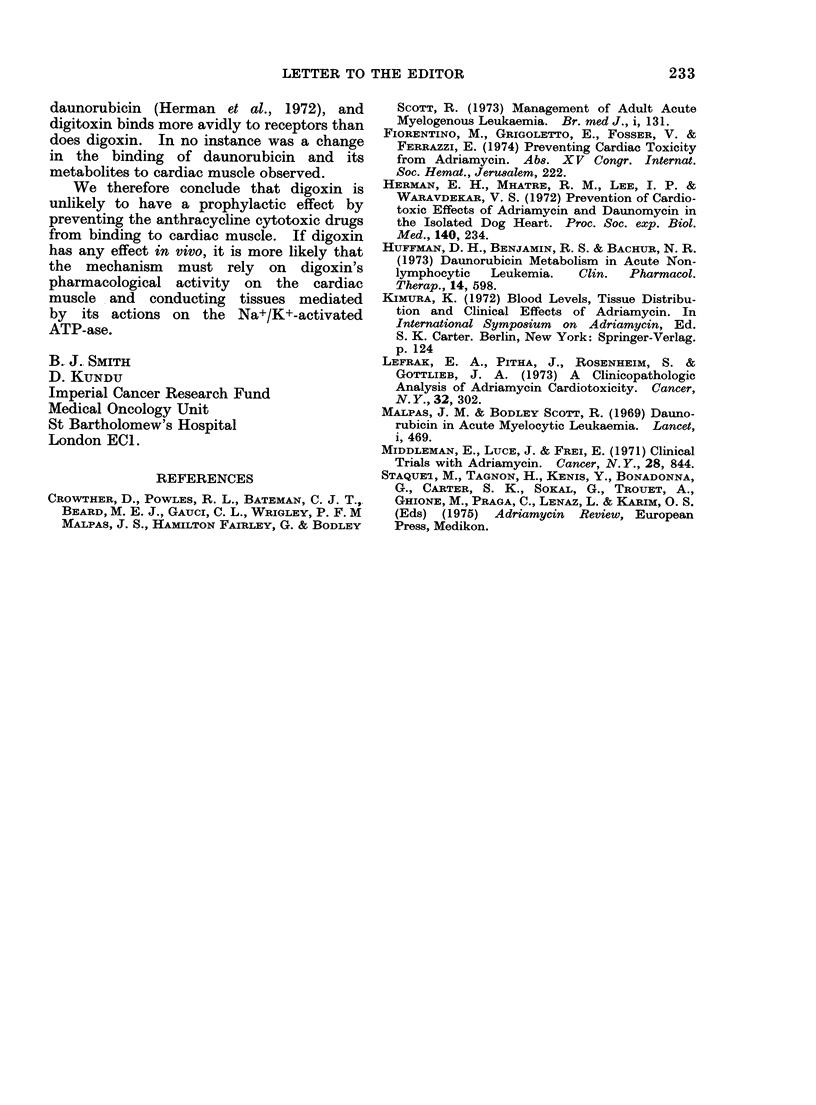# Letter: Digoxin does not prevent daunorubicin or adriamycin from binding to rat heart muscle.

**DOI:** 10.1038/bjc.1976.30

**Published:** 1976-02

**Authors:** B. J. Smith, D. Kundv


*Br. J. Cancer* (1976) **33**, 232

### *Letter to the Editor*

### DIGOXIN DOES NOT PREVENT DAUNORUBICIN OR ADRIAMYCIN FROM BINDING TO RAT HEART MUSCLE

Daunorubicin and adriamycin are anthracycline drugs used in the chemotherapy of acute myelogenous leukaemia and other cancers (Crowther *et al.*, 1973; Staquet *et al.*, 1975). Their use is limited by their tendency to induce cardiac failure (Middleman, Luce and Frei, 1971; Malpas and Bodley Scott 1969), but there have been a number of reports (Lefrak *et al.*, 1973; Kimura, 1972) to suggest that these cardiotoxic effects may be attenuated by the prior administration of digoxin. The mechanism of this reported attenuation is unknown, but it has been proposed (Fiorentino *et al.*, 1974) that perhaps the cardiac glycoside digoxin competes with the anthracycline glycosides for the same binding sites. We have examined this possibility in the rat but have found no evidence to suggest that prior treatment with digoxin can prevent adriamycin and daunorubicin binding to rat cardiac muscle.

We used adult male Wistar rats, each weighing approximately 250 g. Sets of 4 rats were injected intravenously with up to 500 μg digoxin, or alternatively by 3 daily injections of 500 μg digoxin. At various times after injection the rats were injected with various amounts of daunorubicin; up to 20 mg per rat. At 0, 4, 8 and 24 h after daunorubicin injection a rat was killed, its heart removed, washed in ice-cold isotonic saline, minced, homogenized in 5 ml ice-cold saline and the bound daunorubicin and its metabolites extracted according to the method of Huffman, Benjamin and Bachur (1973). This method involves extraction with an equal volume of 75% ethanol, 0·45 N HCl; removal of the precipitate by centrifugation; concentrating the supernatant under nitrogen, and chromatography of the resulting solution on thin layer chromatography. In each case the entire extract from one heart was spotted on Silica gel 60 (without fluorescent indicator) (Merck, Darmstadt) and the chromatograms developed using Solvent system I ($CHCl_3$ $CH_3OH$:$CH_3COOH$;: 100:2: 2.5). No matter what the ratio of injected digoxin to daunorubicin, the pattern of metabolites is identical to that extracted from a control rat receiving only daunorubicin. This was confirmed quantitatively by measuring the eluted spots in a fluorimeter. A typical experiment is shown in the Table.

Similar experiments have been performed with drugs other than daunorubicin. We have used adriamycin, propranolol (4 mg/250 g rat), ICRF 159 (100 mg/rat), and digitoxin (500 μg/rat) instead of digoxin. Propranolol binds to beta-adrenergic receptors and might therefore compete with daunorubicin if it were to bind to the beta receptors on the heart. ICRF 159 has been reported to afford some prophylactic protection against

TABLE.—*Fluorimeter Readings of Eluted Spots: excited at* 474 *nm, Fluorescence read at* 550 *nm on an Amico-Bowman Spectrophotofluorimeter. In this Experiment the Rats received* 200 μg *Daunorubicin* 3 *h after receiving Saline or* 500 μg *Digoxin. Rats were Killed* 2 *h Afterwards or* 24 *h Afterwards*

| Spot | | Rat killed 2 h after daunorubicin injection | | Rat killed 24 h after daunorubicin injection | |
|---|---|---|---|---|---|
| $R_f$ | Colour | No digoxin | 500 μg digoxin | No digoxin | 500 μg digoxin |
| 0·22 | red | 0·012 | 0·011 | 0·011 | 0·013 |
| 0·49 | red | 0·021 | 0·018 | 0·019 | 0·019 |
| *0·53 | red | 0·056 | 0·057 | 0·059 | 0·058 |
| 0·71 | yellow | 0·007 | 0·009 | 0·009 | 0·008 |
| Background | – | 0·001 | 0·001 | 0·001 | 0·001 |

* This spot has the same $R_f$ as the aglycone of daunorubicin in our experiments.

daunorubicin (Herman *et al.*, 1972), and digitoxin binds more avidly to receptors than does digoxin. In no instance was a change in the binding of daunorubicin and its metabolites to cardiac muscle observed.

We therefore conclude that digoxin is unlikely to have a prophylactic effect by preventing the anthracycline cytotoxic drugs from binding to cardiac muscle. If digoxin has any effect *in vivo*, it is more likely that the mechanism must rely on digoxin's pharmacological activity on the cardiac muscle and conducting tissues mediated by its actions on the $Na^+/K^+$-activated ATP-ase.

B. J. SMITH
D. KUNDU
Imperial Cancer Research Fund
Medical Oncology Unit
St Bartholomew's Hospital
London EC1.

### REFERENCES

CROWTHER, D., POWLES, R. L., BATEMAN, C. J. T., BEARD, M. E. J., GAUCI, C. L., WRIGLEY, P. F. M MALPAS, J. S., HAMILTON FAIRLEY, G. & BODLEY SCOTT, R. (1973) Management of Adult Acute Myelogenous Leukaemia. *Br. med J.*, i, 131.

FIORENTINO, M., GRIGOLETTO, E., FOSSER, V. & FERRAZZI, E. (1974) Preventing Cardiac Toxicity from Adriamycin. *Abs. XV Congr. Internat. Soc. Hemat., Jerusalem*, 222.

HERMAN, E. H., MHATRE, R. M., LEE, I. P. & WARAVDEKAR, V. S. (1972) Prevention of Cardiotoxic Effects of Adriamycin and Daunomycin in the Isolated Dog Heart. *Proc. Soc. exp. Biol. Med.*, **140**, 234.

HUFFMAN, D. H., BENJAMIN, R. S. & BACHUR, N. R. (1973) Daunorubicin Metabolism in Acute Nonlymphocytic Leukemia. *Clin. Pharmacol. Therap.*, **14**, 598.

KIMURA, K. (1972) Blood Levels, Tissue Distribution and Clinical Effects of Adriamycin. In *International Symposium on Adriamycin*, Ed. S. K. Carter. Berlin, New York: Springer-Verlag. p. 124

LEFRAK, E. A., PITHA, J., ROSENHEIM, S. & GOTTLIEB, J. A. (1973) A Clinicopathologic Analysis of Adriamycin Cardiotoxicity. *Cancer, N.Y.*, **32**, 302.

MALPAS, J. M. & BODLEY SCOTT, R. (1969) Daunorubicin in Acute Myelocytic Leukaemia. *Lancet*, i, 469.

MIDDLEMAN, E., LUCE, J. & FREI, E. (1971) Clinical Trials with Adriamycin. *Cancer, N.Y.*, **28**, 844.

STAQUET, M., TAGNON, H., KENIS, Y., BONADONNA, G., CARTER, S. K., SOKAL, G., TROUET, A., GHIONE, M., PRAGA, C., LENAZ, L. & KARIM, O. S. (Eds) (1975) *Adriamycin Review*, European Press, Medikon.